# An investigation of the effects of *BMPR1B*, *BMP15*, and *GDF9* genes on litter size in Ramlıç and Dağlıç sheep

**DOI:** 10.5194/aab-64-223-2021

**Published:** 2021-06-03

**Authors:** Koray Çelikeloğlu, Mustafa Tekerli, Metin Erdoğan, Serdar Koçak, Özlem Hacan, Zehra Bozkurt

**Affiliations:** 1 Department of Animal Science, Faculty of Veterinary Medicine, Afyonkarahisar, Turkey; 2 Department of Veterinary Biology and Genetics, Faculty of Veterinary Medicine, Afyonkarahisar, Turkey

## Abstract

This study was carried out to determine the presence of polymorphisms in genes affecting litter size. The SNPs in bone morphogenetic protein receptor type 1B (*BMPR1B*), bone morphogenetic protein 15 (*BMP15*), and growth differentiation factor 9 (*GDF9*) genes were detected in 60 uniparous and 60 multiparous ewes from Ramlıç and Dağlıç breeds. The ewes are maintained in nine public herds at the breeding station of the Afyonkarahisar Sheep and Goats Breeders' Association and lambed in two consecutive breeding seasons. PCR and DNA sequencing analyses were conducted, and 36, 4, and 11 SNPs in Ramlıç and 40, 3, and 11 SNPs in Dağlıç were detected in *BMPR1B*, *BMP15*, and *GDF9* genes, respectively. A total of 16 SNPs in Ramlıç and 10 SNPs in Dağlıç breeds for three genes were found to be significant (P<0.05). The resulting analyses showed that four SNPs (g.49496G>A, c.1658A>C, c.2037C>T, c.2053C>T) of the *BMPR1B* gene and one deletion mutation (c.28_30delCTT) in the *BMP15* gene of the Ramlıç breed as well as five SNPs (c.1487C>A, c.2492C>T, c.2523G>A, c.2880A>G, and c.2763G>A) of the *BMPR1B* gene of the Dağlıç breed have significant positive regression coefficients in the desired direction of the rare allele. The observed mutations have potential to be used as genetic markers in the selection of prolific animals for both breeds.

## 1 Introduction

Crossbreeding with various foreign breeds is performed worldwide to increase the yield of native sheep breeds. Ramlıç sheep were developed by crossbreeding American-originated Rambouillet rams with native Dağlıç ewes (Akçapınar, 2000). The Dağlıç sheep is one of the oldest autochthonous breeds in Asia Minor and has existed since the ancient era. The abilities of Dağlıç to resist the harsh environment, as well as its superior meat and fleece characteristics of the Rambouillet, were combined with the composite breed Ramlıç, carrying 65 %–70 % Rambouillet and 30 %–35 % Dağlıç genotypes. The average lamb birth weight and weaning weight in Ramlıç and Dağlıç breeds were reported as 4.26 and 30.39 as well as 3.54 and 23.25 kg, respectively. These figures hit 80 and 123 as well as 46 and 53 kg in mature ewes and rams in public herds. In addition, the litter size was reported between 1.23 and 1.56 and between 1.02 and 1.04 per ewe, respectively (Akçapınar, 2000; Koçak et al., 2016; Soysal, 2010; Tekerli et al., 2016; Yalçın, 1982).

The profitability of meat type from sheep breeding depends on the weight and litter size. Therefore, selection usually concentrates on these characteristics (Kumm, 2009; Notter, 2008; Tosh and Kemp, 1994). Due to low heritabilities of fertility-related traits, using molecular techniques and combining the results with numerical data is becoming important in this field (Pramod et al., 2013). The fact that economic conditions require rapid genetic progress makes it necessary to choose viable animals. In order to detect genetic variation in animal populations, DNA-level studies would give more reliable results, and many methods such as SNP, microsatellites, RFLP, and microarrays are used for these purposes. It was reported that SNPs have a greater potential than the others for realizing genetic progress (Pramod et al., 2013).

Genetic mutations in fecundity genes can change ovulation rate and litter size (Karsli et al., 2012; Mahdavi et al., 2014). Some of these are *BMPR1B*, *BMP15*, and *GDF9* genes belonging to the super family of transforming growth factor β (TGFβ) obtained from the ovary (Drouilhet et al., 2013). These three genes, which are closely related to each other, have a significant impact on the ovulation rate and litter size (Pramod et al., 2013). These multiple birth genes belonging to the TGFβ superfamily are dominant, and the mutation of a single allele is sufficient to change the ovulation rate and litter size (Davis, 2005; Heaton et al., 2017). *BMPR1B* is on chromosome 6 and encodes the bone morphogenetic protein 1B receptor in the ovary (Davis, 2005). The mutation (FecB or Booroola) in this gene has a role in multiple birth. Australian Booroola Merino is one of the prolific breeds carrying this mutation. It is developed by crossing Merino and Garole sheep (Davis et al., 1982). This type of mutation is also common to some Asian sheep breeds such as Chinese small-tailed Han and Hu sheep, Indian Garole and Kendrapada sheep, Iranian Kalehkoohi sheep, and Indonesian thin-tailed Javanese sheep (Davis et al., 2002, 2006; Fogarty, 2009; Mahdavi et al., 2014; Mulsant et al., 2001; Tang et al., 2018). *BMP15*, also known as the *GDF9B* (FecX) gene, is on the X chromosome and encodes bone morphogenetic protein 15, playing an important role in follicular development in sheep (Hanrahan et al., 2004). The same gene causing prolificacy in heterozygous Romneys is called the Inverdale gene (FecX${}^{I}$). The mutations of this gene identified in various breeds are called by different names such as FecX${}^{I}$, FecX${}^{H}$, FecX${}^{G}$, FecX${}^{B}$, FecX${}^{L}$, FecX${}^{R}$, FecX${}^{\text{Gr}}$, and FecX${}^{O}$ (Alink et al., 2005; Davis, 2005; Davis et al., 1999). The *GDF9* (FecG) gene on the fifth chromosome of sheep is also known to affect litter size and ovulation rate. Eight different point mutations (G1–G8) were detected on this gene. Ewes carrying a single copy of the FecG in Cambridge and Belclare sheep breeds had a 1.4 times higher ovulation rate (Davis, 2004, 2005).

This study aimed to determine the possible associations of mutations in *BMPR1B*, *BMP15*, and *GDF9* genes with the litter size in Ramlıç and Dağlıç sheep and investigate the possibilities of using these relationships in a selection program.

## 2 Materials and methods

### 2.1 Localization and animal material

The study was carried out in the Emirdağ (39${}^{\circ }$1${}^{\prime }$1${}^{\prime \prime }$ N, 31${}^{\circ }$9${}^{\prime }$0${}^{\prime \prime }$ E, 970 m) and Bolvadin (38${}^{\circ }$42${}^{\prime }$40${}^{\prime \prime }$ N, 31${}^{\circ }$2${}^{\prime }$55${}^{\prime \prime }$ E, 995 m) districts of the Afyonkarahisar province in Turkey. The research was conducted in nine enterprises within the scope of the “National Community-Based Small Ruminant Improvement” project coordinated by the General Directorate of Agricultural Research and Policies of the Ministry of Agriculture and Forestry. In these farm operations, sheep were fed on a pasture basis, and the additional concentrate and roughage supply was provided in times of feed shortage. A total of 30 uniparous and 30 multiparous ewes per breed (Ramlıç and Dağlıç), lambing in two consecutive breeding seasons, were used. An ethical committee approval was obtained from the Afyon Kocatepe University Animal Experiments Local Ethics Committee with decision no. 49533702/18 dated 25 February 2016.

### 2.2 Sample collection and DNA isolation

Blood samples were collected from the *V. jugularis* of the animals and then placed into vacuum tubes with EDTA. The blood samples were brought to the laboratory in a cold chain, kept at +4 ${}^{\circ }C$ until the DNA isolation stage, and then stored at -20 ${}^{\circ }C$ in cryotubes. Genomic DNA was obtained using a commercial DNA isolation kit (Thermo K0722). DNA samples were checked for integrity with a 0.6 % agarose gel; the amount and quality were measured using spectrophotometer devices (Multiscan GO and Qubit). Subsequently, the DNA samples were adjusted as 20 $\mathrm{ng}\;µL^{-1}$.

### 2.3 PCR and DNA sequencing analysis

Primers prepared with the FastPCR 6.0 package program (Kalendar et al., 2009) given in Table 1 were used for PCR analysis. Primers were designed by using intronic parts to sequence the related exons.

**Table 1:** Genes affecting multiple births, size, and $T_{m}$ degrees.

Gene		Forward ($5^{\prime }\to 3^{\prime }$)	Reverse ($5^{\prime }\to 3^{\prime }$)	$T_{m}$ (${}^{\circ }$C)	Size (bp)
*BMPR1B*	Exon 9	TGTGTCTGCTGTATTGGCACAC	GCTAGGAAACCCTGAACATCG	59	419
	Exon 10	AGACACCTATGACAAAGGACG	GCACACAATCCCAGACATTAGC	59	696
	Exon 13${}^{a}$	GTATCGAGTGCCAGCCTTGCA	TCCCACATCCTCTGAAGCTGC	63	852
	Exon 13${}^{b}$	TCTGGGGATTCCCACCCATGAC	GCAAAGAAGAGGGGCTTCCCAA	59	957
*BMP15*	Exon 1	ACATGTTGCTGAACACCAAGC	AGGCAATGTGAAGCCTGACA	63	462
*GDF9*	Exon 1	GAATTGAACCTAGCCCACCCAC	AGCCTACATCAACCCATGAGGC	63	708
	Exon 2	AGGAACCTTTCCATCAGTGGA	TCCTCCCAAAGGCATAGACAGG	58	1043

The PCR mixture of 25 µL is comprised of 2 µL of DNA (20 $\mathrm{ng}\;µ\;L^{-1}$), 1.25 µL of each primer (10 pmol), 10×Q5 reaction buffer 2.5 µL, 0.5 µL (10 mM, Roche) of dNTP mixture, Q5 high-fidelity Taq polymerase (NEB) 0.25 µL, and 18.5 µL of ${\mathrm{ddH}}_{2}O$. The reactions were carried out on the ABI Veriti PCR instrument. The PCR apparatus was programmed at 98 ${}^{\circ }C$ for 30 s: 35 cycles in 10 s at 98 ${}^{\circ }C$, $T_{m}$ (melting temperature) 30 s, 30 s at 72 ${}^{\circ }C$, and 10 min at 72 ${}^{\circ }C$ in the last phase. 5 µL of the PCR product, 0.5 µL Exonuclease-I (Thermo, EN0582), and 1.0 µL FastAP (Thermo, EF0652) were added into the PCR tube to clean the PCR products. The prepared mixture was placed into the PCR and adjusted to 15 min at 37 ${}^{\circ }C$ and 15 min at 85 ${}^{\circ }C$. For DNA sequencing analysis, a total of 20 µL consisting of 2 µL from BigDye Terminator v3.1, 11 µL of 1×sequencing buffer, 5 µL of forward or reverse primer (1 pmol), and 2 µL of cleaned PCR product were prepared, and the PCR was programmed with the purpose of pre-denaturation at 96 ${}^{\circ }C$ for 30 s, separation at 96 ${}^{\circ }C$ for 10 s, adhesion at 54 ${}^{\circ }C$ for 15 s, and extension at 60 ${}^{\circ }C$ for 4 min. The sequence PCR products were then cleaned according to the company's protocol with BigDye XTerminator (Applied Biosystems) and placed in the DNA sequencing device (ABI 3500) after a short centrifuge. Each sample was arranged with Sequencher 5.4.6 (Gene Code Corporation, Ann Arbor, Michigan, USA) and aligned with BioEdit 7.0.9 Sequence Alignment software (Hall, 1999) after the DNA sequencing analysis. Furthermore, the nucleotide sequences determined in the *BMPR1B*, *BMP15*, and *GDF9* genes were aligned with the reference sequences from the NCBI (NM_001009431.1, NM_001114767.1, and NM_001142888.2), and the sequences were translated and compared with the amino acid sequences of the references (NP_ 001009431.1, NP_001108239.1, and NP_ 001136360.2) in BioEdit.

### 2.4 Statistical analyses

The model for analyzing the associations between litter size and SNPs in each breed was $Y_{ijk}=\mu +H_{i}+A_{j}+bX_{ijk}+e_{ijk}$, where the variants of the SNPs are considered covariates. In this equation $Y_{ijk}$ indicates litter size, μ is the overall mean, H is the fixed effect of the herd, A is the random individual effect, b is the linear regression coefficient between the variants of the SNPs and litter size, and $e_{ijk}$ is random error N (0, $\sigma^{2}$). According to Aulchenko et al. (2007) and Khatib (2015) this model takes SNPs as the covariate. Genotypes are coded as 0, 1, and 2 for none, a single copy, and double copies according to the presence of the rare allele. A pedigree file was produced using the records of 70310 individuals. Unpublished software called Pedigri Yıldızı (Tekerli, 2018) was used to create the file. WOMBAT (Meyer and Tier, 2012) software and the run option –snapp were used in the analysis of the model, including SNP effects.

## 3 Results

Different regions of *BMPR1B*, *BMP15*, and *GDF9* genes were examined in the blood samples from Ramlıç and Dağlıç breeds. The populations were found to be in Hardy–Weinberg equilibrium (P<0.05) with a total heterozygosity of 0.1491 and 0.1638.

### 3.1 *BMPR1B* gene

As a result of PCR analysis, PCR products with the size of 419 bp (base pair) at exon 9, 696 bp at exon 10, and 1809 bp at exon 13 in the *BMPR1B* gene were obtained. The distributions of 36 SNPs determined in the *BMPR1B* gene of the Ramlıç breed at the end of the DNA sequencing analysis were as follows: four SNPs in intron 8, three SNPs in exon 9, one SNP in intron 9, two SNPs in exon 10, four SNPs in intron 10, two SNPs in intron 12, and one SNP in exon 13 and 19 in the $3^{\prime }$ UTR. The g.49480C>T, g.49496G>A, c.1658A>C, c.1875T>C, c.1890C>T, c.2037C>T, c.2053C>T, c.2070G>C, c.2083C>T, c.2129C>T, c.2492C>T, c.2523G>A, c.2763G>A, and c.2978C>T were statistically significant (P<0.05) SNPs detected in the 12th intron, 13th exon, and $3^{\prime }$ UTR regions (Table 2).

**Table 2:** The SNPs located in the 12th intron, 13th exon, and $3^{\prime }$ UTR region in the *BMPR1B* gene of Ramlıç sheep, along with their significance levels and calculated regression coefficients.

SNP	Genotype	N‡	Regression coefficient	Standard error	t value	P value related to t value
g.49480C>T	CC	17	$-0.0609240^{b}$	$0.184471\times 10^{-1}$	-3.30264	0.002886323
	CT	17				
	TT	13				
g.49496G>A	GG	37	$0.0827586^{b}$	$0.275030\times 10^{-1}$	3.00908	0.005907815
	AG	8				
	AA	2				
c.1658A>C	AA	40	$0.131989^{b}$	$0.332543\times 10^{-1}$	3.96908	0.00053637
	AC	6				
	CC	1				
c.1875T>C	TT	40	$-0.131989^{b}$	$0.332543\times 10^{-1}$	3.96908	0.00053637
	CT	6				
	CC	1				
c.1890C>T	CC	40	$-0.131989^{b}$	$0.332543\times 10^{-1}$	3.96908	0.00053637
	CT	6				
	TT	1				
c.2037C>T	CC	33	$0.0903010^{b}$	$0.317610\times 10^{-1}$	2.84314	0.008772616
	CT	14				
c.2053C>T	CC	41	$0.145161^{b}$	$0.348742\times 10^{-1}$	4.16243	0.00032614
	CT	5				
	TT	1				
c.2070G>C	CC	41	$-0.145161^{b}$	0.145161	4.16243	0.00032614
	CG	5				
	GG	1				
c.2083C>T	CC	46	$-0.281250^{b}$	$0.970857\times 10^{-1}$	-2.89693	0.007723889
	CT	1				
c.2129C>T	CC	40	$-0.0752284^{a}$	$0.328709\times 10^{-1}$	-2.2886	0.030824655
	CT	6				
	TT	1				
c.2492C>T	TT	38	$-0.137825^{b}$	$0.339816\times 10^{-1}$	-4.05589	0.000429144
	CT	8				
	CC	1				
c.2523G>A	GG	38	$-0.137825^{b}$	$0.339816\times 10^{-1}$	-4.05589	0.000429144
	AG	8				
	AA	1				
c.2763G>A	GG	38	$-0.137825^{b}$	$0.339816\times 10^{-1}$	-4.05589	0.000429144
	AG	8				
	AA	1				
c.2978C>T	CC	44	$-0.207493^{b}$	$0.589651\times 10^{-1}$	-3.51891	0.001683673
	CT	3				

${}^{a}$ P<0.05; ${}^{b}$ P<0.01; $N^{‡}$: number of sheep carrying related genotype.

The composition of 40 SNPs determined in the *BMPR1B* gene of the Dağlıç breed was as follows: five SNPs in intron 8, three SNPs in exon 9, one SNP in intron 9, two SNPs in exon 10, five SNPs in intron 10, two SNPs in intron 12, and one SNP in exon 13 and 21 in the $3^{\prime }$ UTR. The c.1487C>A, c.1658A>C, c.1659T>A, c.1875T>C, c.1890C>T, c.2053C>T, c.2070G>C, c.2880A>G, and c.2978C>T were statistically significant (P<0.05) SNPs detected in the 13th exon and $3^{\prime }$ UTR regions (Table 3).

**Table 3:** The SNPs located in the 13th exon and $3^{\prime }$ UTR region in the *BMPR1B* gene of the Dağlıç breed, along with their significance levels and calculated regression coefficients.

SNP	Genotype	N‡	Regression coefficient	Standard error	t value	P value related to t value
c.1487C>A	CC	28	$0.0559284^{b}$	$0.202225\times 10^{-1}$	2.76566	0.009626563
	AC	20				
	AA	6				
c.1658A>C	AA	44	$-0.0940439^{b}$	$0.338538\times 10^{-1}$	-2.77794	0.00934298
	AC	10				
c.1659T>A	TT	44	$-0.0940439^{b}$	$0.338538\times 10^{-1}$	-2.77794	0.00934298
	AT	10				
c.1875T>C	TT	44	$-0.0940439^{b}$	$0.338538\times 10^{-1}$	-2.77794	0.00934298
	CT	10				
c.1890C>T	CC	44	$-0.0940439^{b}$	$0.338538\times 10^{-1}$	-2.77794	0.00934298
	CT	10				
c.2053C>T	CC	44	$-0.0940439^{b}$	$0.338538\times 10^{-1}$	-2.77794	0.00934298
	CT	10				
c.2070G>C	CC	44	$-0.0940439^{b}$	$0.338538\times 10^{-1}$	-2.77794	0.00934298
	CG	10				
c.2880A>G	AA	48	0.0980392${}^{a}$	$0.423339\times 10^{-1}$	2.31586	0.027584943
	AG	6				
c.2978C>T	CC	46	$-0.0933707^{a}$	$0.369520\times 10^{-1}$	-2.52681	0.017018393
	CT	8				

${}^{a}$ P<0.05; ${}^{b}$ P<0.01; $N^{‡}$: number of sheep carrying related genotype.

### 3.2 *BMP15* gene

A PCR product with the size of 462 bp was obtained in the first exon of the *BMP15* gene. A total of three SNPs in both breeds were found at the end of DNA sequencing analysis. Two of them were in the first exon and one of them was in the first intron of the *BMP15* gene. It was determined that c.28_30delCTT and c.199C>T in the Ramlıç breed as well as g.383G>A in the Dağlıç breed were statistically significant (P<0.05) SNPs detected in the first exon and first intron (Tables 4 and 5).

**Table 4:** The SNPs located in the first exon and intron in the *BMP15* gene of the Ramlıç breed, with their significance levels and calculated regression coefficients.

SNP	Genotype	N‡	Regression coefficient	Standard error	t value	P value related to t value
c.28_30delCTT	CTTCTT	32	$0.117449^{*}$	$0.216316\times 10^{-1}$	5.42949	0.0000018409
	CTTdel	15				
	deldel	4				
c.199C>T	CC	50	$-0.272727^{*}$	0.0970411	-2.81043	0.0071401619
	CT	1				

${}^{*}$ P<0.01; $N^{‡}$: number of sheep carrying related genotype.

**Table 5:** The SNPs located in the first exon and intron in the *BMP15* gene of the Dağlıç breed, with their significance levels and calculated regression coefficients.

SNP	Genotype	N‡	Regression coefficient	Standard error	t value	P value related to t value
g.383G>A	GG	51	$-0.135135^{*}$	0.0573907	-2.3547	0.022591054
	GA	3				

${}^{*}$ P<0.05; $N^{‡}$: number of sheep carrying related genotype.

### 3.3 *GDF9* gene

PCR products with the size of 708 bp in exon 1 and 1043 bp in exon 2 were obtained in the *GDF9* gene. At the end of the DNA sequencing analysis, a total of 11 SNPs were obtained; two of them were in exon 1, two in intron 1, and seven in exon 2 of the *GDF9* gene of the Ramlıç breed. A total of 11 SNPs were also obtained in the Dağlıç breed; two were in the first exon, one in the first intron, and eight in the second exon. The regression coefficients of SNPs in this group were not statistically significant.

## 4 Discussion

### 4.1 *BMPR1B* gene

The findings of this study showed that SNPs detected in the *BMPR1B* gene were consistent with the results in the literature for Booroola Merino, Han and Hu, Kendrapada, and Javanese sheep (Davis et al., 2002, 2006; Fogarty, 2009; Heaton et al., 2017). The effects of SNPs located in intron 8, exon and intron 9, and exon and intron 10 were not statistically significant in either breed. The non-significant results may have originated from the methods of the research, the breeds, and/or the models used in statistical evaluations. No literature studies reporting significant effects of these SNPs were encountered. FecB is a point mutation at the nucleotide position 830, which results in the replacement of the glutamine-to-arginine amino acid (Q249R) (Souza et al., 2001). This mutation was not able to be detected in the present study. However, 22 new SNPs with statistical significance were identified. The significant and positive regression coefficients for SNPs of Ramlıç (g.49496G>A, c.1658A>C, c.2037C>T and c.2053C>T) and Dağlıç (c.1487C>A, c.2492C>T, c.2523G>A, c.2880A>G, and c.2763G>A) breeds showed that the individuals carrying rare alleles have more potential for multiple births.

### 4.2 *BMP15* gene

A total of four SNPs were found in the *BMP15* gene: three in the first exon and one in the first intron. Similar results indicating that different mutations may be encountered in the gene were reported by different researchers (Davis, 2005; Demars et al., 2013; Drouilhet et al., 2013; Gürsel, 2009; Monteagudo et al., 2009) in Romney, Belclare, Cambridge, Lacaune, Rasa Aragonesa, Grivette, and Olkuska sheep. The c.28_30delCTT mutation with low frequencies detected in exon 1 of this gene of the Ramlıç breed causes the 10th amino acid (leucine) not to take part in protein synthesis. Different researchers (Demars et al., 2013; Hanrahan et al., 2004) have found similar results, determining the c.28_30delCTT mutation in Cambridge, Belclare, Olkuska, and Grivette sheep, but alleged that it was not functional in the occurrence of multiple births. However, it was highly significant (P<0.000001) in this study, and the direction of the regression line showed that the rare allele can increase the rate of multiple births. The SNP (c.199C>T) in Ramlıç and the SNP (g.383G>A) in Dağlıç breeds were also significant, but the regression coefficients were negative. Therefore, the selection for wild-type alleles will be more beneficial in sheep improvement programs. No literature studies on mutations, other than the c.28_30delCTT deletion mutation detected in the *BMP15* gene, have been encountered.

### 4.3 *GDF9* gene

A total of 13 SNPs were detected in the gene: two in the first exon, two in the first intron, and nine in the second exon in both Ramlıç and Dağlıç breeds. This finding is consistent with the results reported by different researchers (Davis, 2004, 2005; Demars et al., 2013; Hanrahan et al., 2004; Våge et al., 2013) in Cambridge, Belclare, Santa Inês, and Norway White sheep. Hanrahan et al. (2004) reported that c.260G>A, c.471C>T, c.477G>A, c.721G>A, c.978A>G, and c.994G>A point mutations were non-functional, in agreement with this study.

## 5 Conclusion

It has been reported in the literature that the litter size is affected by the *BMPR1B*, *BMP15*, and *GDF9* genes. The effects of the first two genes were also significant for both breeds in this study. Accordingly, the regression coefficients of the SNPs for Ramlıç (g.49496G>A, c.1658A>C, c.2037C>T, and c.2053C>T) and Dağlıç breeds (c.1487C>A, c.2492C>T, c.2523G>A, c.2880A>G, and c.2763G>A) for the *BMPR1B* gene were statistically significant and in the desired direction of rare alleles. Significant mutation of c.28_ 30delCTT in the Ramlıç breed for *BMP15* showed that the rare alleles may be functional. The SNP differences in the two breeds showed that crossbreeding (Rambouillet with Dağlıç) increased the allelic diversity in Ramlıç sheep. Polymorphisms found in both breeds would be beneficial in selection programs for producing more prolific animals.

## Supplement

10.5194/aab-64-223-2021-supplement The supplement related to this article is available online at: https://doi.org/10.5194/aab-64-223-2021-supplement.

## Data Availability

The data from this study can be obtained from the authors upon reasonable request.

## Code availability

WOMBAT software is available at (last access: June 2021) (REML, 2021).
