# Supplementary material for: An investigation of the effects of BMPR1B, BMP15, and GDF9 genes on litter size in Ramlıç and Dağlıç sheep
Source: Arch Anim Breed. 2021 Jun 3;64(1):223–30. doi: 10.5194/aab-64-223-2021 (PMC8209503; doi:10.5194/aab-64-223-2021)
Supplement: The supplement related to this article is available online at: https://doi.org/10.5194/aab-64-223-2021-supplement. [file aab-64-223-supplement.pdf]

Supplement of Arch. Anim. Breed., 64, 223–230, 2021
https://doi.org/10.5194/aab-64-223-2021-supplement
© Author(s) 2021. CC BY 4.0 License.

Open Access
Archives Animal Breeding

Supplement of

An investigation of the effects of *BMPR1B*, *BMP15*, and *GDF9* genes on litter size in Ramlıç and Dağlıç sheep

Koray Çelikeloğlu et al.

Correspondence to: Koray Çelikeloğlu (kcelikeloglu@aku.edu.tr) and Mustafa Tekerlı (tekerli@aku.edu.tr)

The copyright of individual parts of the supplement might differ from the article licence.

Table S1

Gene		Forward (5'→3')	Reverse (5'→3')	Tm (°C)	Size (bp)
**BMPR1B**	Exon 9	TGTGTCTGCTGTATTGGCACAC	GCTAGGAAACCCTGAACATCG	59	419
	Exon 10	AGACACCTATGACAAAGGACG	GCACACAATCCCAGACATTAGC	59	696
	Exon 13[a]	GTATCGAGTGCCAGCCTTGCA	TCCCACATCCTCTGAAGCTGC	63	852
	Exon 13[b]	TCTGGGGATTCCCACCCATGAC	GCAAAGAAGAGGGGCTTCCCAA	59	957
**BMP15**	Exon 1	ACATGTTGCTGAACACCAAGC	AGGCAATGTGAAGCCTGACA	63	462
**GDF9**	Exon 1	GAATTGAACCTAGCCCACCCAC	AGCCTACATCAACCCATGAGGC	63	708
	Exon 2	AGGAACCTTTCCATCAGTGGA	TCCTCCCAAAGGCATAGACAGG	58	1043

Table S2

SNP	GENOTYPE	N‡	Regression Coefficient	Standard Error	t- value	P-value related to t-value
g.49480C>T	CC	17	-0.0609240**	0.184471E-01	-3.30264	0.002886323
	CT	17				
	TT	13				
g.49496G>A	GG	37	0.0827586**	0.275030E-01	3.00908	0.005907815
	AG	8				
	AA	2				
c.1658A>C	AA	40	0.131989**	0.332543E-01	3.96908	0.00053637
	AC	6				
	CC	1				
c.1875T>C	TT	40	-0.131989**	0.332543E-01	3.96908	0.00053637
	CT	6				
	CC	1				
c.1890C>T	CC	40	-0.131989**	0.332543E-01	3.96908	0.00053637
	CT	6				
	TT	1				
c.2037C>T	CC	33	0.0903010**	0.317610E-01	2.84314	0.008772616
	CT	14				
c.2053C>T	CC	41	0.145161**	0.348742E-01	4.16243	0.00032614
	CT	5				
	TT	1				
c.2070G>C	CC	41	-0.145161**	0.145161	4.16243	0.00032614
	CG	5				
	GG	1				
c.2083C>T	CC	46	-0.281250**	0.970857E-01	-2.89693	0.007723889
	CT	1				
c.2129C>T	CC	40	-0.0752284*	0.328709E-01	-2.2886	0.030824655
	CT	6				
	TT	1				
c.2492C>T	TT	38	-0.137825**	0.339816E-01	-4.05589	0.000429144
	CT	8				
	CC	1				
c.2523G>A	GG	38	-0.137825**	0.339816E-01	-4.05589	0.000429144
	AG	8				
	AA	1				
c.2763G>A	GG	38	-0.137825**	0.339816E-01	-4.05589	0.000429144
	AG	8				
	AA	1				
c.2978C>T	CC	44	-0.207493**	0.589651E-01	-3.51891	0.001683673
	CT	3				

*P<0.05 ** P<0.01 N‡: number of sheep carrying related genotype

Table S3

SNP	GENOTYPE	N‡	Regression Coefficient	Standard Error	t- value	P-value related to t-value
c.1487C>A	CC	28	0.0559284**	0.202225E-01	2.76566	0.009626563
	AC	20				
	AA	6				
c.1658A>C	AA	44	-0.0940439**	0.338538E-01	-2.77794	0.00934298
	AC	10				
c.1659T>A	TT	44	-0.0940439**	0.338538E-01	-2.77794	0.00934298
	AT	10				
c.1875T>C	TT	44	-0.0940439**	0.338538E-01	-2.77794	0.00934298
	CT	10				
c.1890C>T	CC	44	-0.0940439**	0.338538E-01	-2.77794	0.00934298
	CT	10				
c.2053C>T	CC	44	-0.0940439**	0.338538E-01	-2.77794	0.00934298
	CT	10				
c.2070G>C	CC	44	-0.0940439**	0.338538E-01	-2.77794	0.00934298
	CG	10				
c.2880A>G	AA	48	0.0980392*	0.423339E-01	2.31586	0.027584943
	AG	6				
c.2978C>T	CC	46	-0.0933707*	0.369520E-01	-2.52681	0.017018393
	CT	8				

*P<0.05 ** P<0.01 N‡: number of sheep carrying related genotype

Table S4

SNP	GENOTYPE	N‡	Regression Coefficient	Standard Error	t- value	P-value related to t-value
c.28_30delCTT	CTTCTT	32	0.117449**	0.216316E-01	5.42949	0.0000018409
	CTTdel	15				
	deldel	4				
c.199C>T	CC	50	-0.272727**	0.0970411	-2.81043	0.0071401619
	CT	1				

** P<0.01 N‡: number of sheep carrying related genotype

Table S5

SNP	GENOTYPE	N‡	Regression Coefficient	Standard Error	t- value	P-value related to t-value
g.383G>A	GG	51	-0.135135*	0.0573907	-2.3547	0.022591054
	GA	3				

* P<0.05 N‡: number of sheep carrying related genotype
